# Perovskite CsPbI_3_ Nanocrystal and Nanowire Incorporated G‐C_3_N_4_ Nanosheets for CO_2_ Reduction and NO Removal

**DOI:** 10.1002/advs.78119

**Published:** 2026-09-30

**Authors:** Xiao Zhang, San Ping Jiang

**Affiliations:** ^1^ Advanced Institute for Materials Research (WPI‐AIMR) Tohoku University Sendai Japan; ^2^ WA School of Mines: Minerals Energy and Chemical Engineering Curtin University Perth Western Australia Australia

**Keywords:** CsPbI_3,_ g‐C_3_N_4_, heterostructures, NO/CO_2_, perovskite, photocatalysis

## Abstract

Expanded light absorption and optimized heterostructure interfaces can significantly enhance photocatalytic redox performance. Herein, perovskite CsPbI_3_ nanocrystals (NCs) and nanowires (NWs) are integrated with graphitic carbon nitride (g‐C_3_N_4_) nanosheets via a combined mechano‐chemical pre‐reaction and solvothermal process. This strategy effectively removes surface ligands and enables the formation of well‐defined heterostructures with strong interfacial contact, facilitating efficient charge carrier transfer. The distinct morphologies and band structures of CsPbI_3_ NCs and NWs lead to differentiated photocatalytic behaviors in CO_2_ reduction and NO removal. An S‐scheme charge transfer mechanism further promotes charge separation and migration. Among the composites, g‐C_3_N_4_/CsPbI_3_ NWs exhibit superior CO_2_ reduction activity, achieving a visible‐light‐driven CO production rate of 4.6 µmol g^−1^ h^−1^, attributed to the face‐to‐face interface and one‐dimensional architecture that enhance charge transport. In contrast, g‐C_3_N_4_/CsPbI_3_ NCs demonstrate excellent NO removal performance, with a removal efficiency of 69.5% and low NO_2_ selectivity (5.0%) at 600 ppb NO, owing to the generation of high‐energy photoinduced charge carriers. This work presents a versatile design strategy for g‐C_3_N_4_‐based photocatalysts toward efficient energy conversion and environmental remediation.

## Graphical abstract

CsPbI_3_ nanostructures integrated with g‐C_3_N_4_ formed S‐scheme heterojunctions enabling efficient photocatalytic CO_2_ reduction and NO abatement. The g‐C_3_N_4_/CsPbI_3_ nanowires achieved a CO production rate of 4.6 µmol g^−1^ h^−1^, while nanocrystal composites delivered 69.5% NO removal with only 5.0% NO_2_ selectivity, driven by highly energetic charge carriers from CsPbI_3_ NCs.

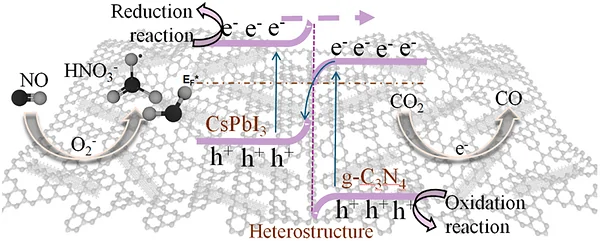

## 1 Introduction

Photocatalytic technologies have been broadly developed to utilize solar energy for both chemical fuel production and environmental pollutant degradation [1, 2]. Graphitic carbon nitride (g‐C_3_N_4_), a prototypical two‐dimensional (2D) layered semiconductor active under visible light (blue light region), has been extensively studied owing to its metal‐free framework and remarkable stability [3, 4]. Despite its advantages, g‐C_3_N_4_ exhibits limited intrinsic absorption mainly within the blue region, rendering heterostructure design crucial for extending its spectral response, enhancing solar utilization, and strengthening the redox capability of photogenerated carriers [5, 6]. Owing to their ultrathin layered architecture and extensive surface area, 2D g‐C_3_N_4_ nanosheets are particularly well‐suited as platforms for assembling semiconductor heterostructures [7, 8]. Considering that ultraviolet light accounts for less than 5% of the total solar spectrum [9], it is highly desirable to couple g‐C_3_N_4_ with semiconductors responsive to visible and near‐infrared light to maximize solar energy harvesting [10]. Furthermore, the interfacial properties and heterostructure configurations play critical roles in determining the efficiency of photogenerated charge carrier separation and transfer. Therefore, the rational design of g‐C_3_N_4_‐based heterostructures with uniformly distributed nanocomponents has emerged as a key research direction for achieving enhanced photocatalytic performance [11].

All inorganic perovskite nanomaterials have been extensively investigated in photovoltaic and luminescence‐related fields, driven by their remarkable optoelectronic performance [12, 13]. Their emission profiles cover nearly the entire visible spectrum, providing tunable absorption from blue to red regions [14, 15]. Moreover, the combination of phase diversity and band gap tunability renders them highly promising for the rational design of photocatalytic heterostructures, thereby driving increasing efforts toward their application in enhanced photocatalysis [16, 17]. As an example, Wang and co‐workers reported silica‐coated CsPbBr_3_ nanocrystals (NCs) integrated with hollow, bowl‐shaped TiO_2_ nanoarrays, which exhibited efficient NO removal [18]. Notably, among perovskite materials, CsPbI_3_ distinguishes itself by its relatively narrow band gap, enabling strong absorption across the red and near‐infrared spectral regions [19, 20, 21]. Despite their promising properties, investigations into CsPbI_3_ NCs coupled with g‐C_3_N_4_ nanosheets are still limited, primarily restricted to a few applications such as dichlorophenol degradation and H_2_O_2_ production [22, 23]. A critical challenge stems from the inherent instability of the CsPbI_3_ phase in polar and humid conditions, originating from the insufficient ionic radius of Cs^+^ to stabilize the [PbI_6_]^4−^ octahedral lattice, thereby triggering structural distortion and phase degradation [24, 25, 26, 27]. Therefore, the development of robust stabilization strategies that preserve the intrinsic optoelectronic advantages of CsPbI_3_ remains a central challenge in enabling its practical deployment in photocatalysis.

The morphology of nanocomponents critically governs both the structure and catalytic behavior of heterostructured systems [28, 29, 30, 31]. For example, one‐dimensional (1D) nanowires (NWs) are particularly advantageous for promoting efficient charge carrier separation and enabling directional charge migration. Meanwhile, all‐inorganic perovskite nanomaterials can be engineered into a wide range of morphologies, such as quantum dots, cubic NCs, NWs, and nanoplates, offering versatile platforms for tuning their physicochemical properties and catalytic functions [13, 15, 24]. Compared with CsPbCl_3_ and CsPbBr_3_, CsPbI_3_ features a narrower and tunable band gap, allowing its optical absorption to extend from the visible into the near‐infrared region. This expanded spectral response significantly improves the solar light harvesting capability of g‐C_3_N_4_, thus promoting more efficient generation and utilization of photogenerated charge carriers [32, 33, 34]. Moreover, the high crystallinity of CsPbI_3_ NCs and 1D NWs enables the formation of intimate face‐to‐face interfaces with g‐C_3_N_4_ nanosheets, which often facilitates efficient charge separation and interfacial transport, thereby enhancing photocatalytic activity. Nevertheless, these CsPbI_3_ nanostructures are commonly synthesized under hydrophobic conditions, leading to the retention of organic capping ligands on their surfaces. Such surface ligands impede effective interfacial contact and obstruct charge transport across the heterojunction. Consequently, developing robust fabrication approaches to construct well‐integrated and ligand‐free CsPbI_3_/g‐C_3_N_4_ heterostructures remains a critical challenge. Prior studies have highlighted the considerable photocatalytic potential of perovskite‐based composites. For instance, CsPbBr_3_ quantum dots possess strong electron‐donating (reduction) capability, making them effective candidates for applications such as photocatalytic CO_2_ reduction [35]. However, self‐assembled TiO_2_/CsPbBr_3_ composites often exhibit limited energy transfer efficiency and poor interfacial stability. To overcome these limitations, ultrathin g‐C_3_N_4_ nanosheets offer abundant nitrogen‐active sites that favor oxidation reactions. Additionally, their large surface area and layered structure facilitate the stabilization of perovskite NCs while promoting efficient charge separation and migration [36]. Furthermore, Ou et al. developed an amino‐assisted anchoring approach to immobilize CsPbBr_3_ quantum dots onto porous g‐C_3_N_4_, which significantly improved the photocatalytic performance toward CO_2_ reduction [37].

Both photocatalytic CO_2_ reduction and NO_x_ removal are important research areas for sustainable environmental and energy applications [38, 39]. Although their reaction pathways differ, both processes rely on the same fundamental mechanism involving the generation, separation, and transport of photogenerated charge carriers [40, 41]. Strengthening the redox capability is essential for achieving high overall efficiency. In CO_2_ reduction, the formation of various carbon‐based products (e.g., C_1_, C_2_, and C_3_ species) introduces an additional challenge, as product selectivity, particularly toward single carbon products, plays a crucial role alongside conversion efficiency. As a result, enhancing selectivity has become a major focus. Similarly, in NO removal, suppressing NO_2_ formation has attracted significant attention because NO_2_ released into the atmosphere often contributes to acid rain. In this study, CsPbI_3_ perovskite NCs and 1D NWs were interfaced with g‐C_3_N_4_ nanosheets to construct tightly coupled face‐to‐face heterostructures, enabling efficient interfacial charge transfer for enhanced photocatalytic CO_2_ reduction and NO removal. The engineered CsPbI_3_/g‐C_3_N_4_ heterostructures exhibited a striking morphology‐dependent bifunctionality, whereby the dimensionality of CsPbI_3_ uniquely dictated the photocatalytic redox pathway. Specifically, integration of CsPbI_3_ NWs with g‐C_3_N_4_ enabled highly efficient CO_2_ reduction, achieving a CO evolution rate of 4.6 µmol g^−1^ h^−1^ under visible light. In sharp contrast, nanocrystal‐based composites demonstrated exceptional NO oxidation performance, reaching 69.5% removal efficiency at 600 ppb with minimal NO_2_ byproduct formation (5.0%). This pronounced functional divergence highlights a previously underexplored strategy of morphology‐governed redox tuning, where nanowires facilitated directional electron transport to promote reduction reactions, while nanocrystals intrinsically favored oxidation pathways due to their distinct electronic structure.

## 2 Results and discussion

Figure 1 illustrates the fabrication pathway for integrating CsPbI_3_ NWs with g‐C_3_N_4_ nanosheets. Detailed experimental procedures are provided in the Experimental Section of the Supporting Information. Ultrathin g‐C_3_N_4_ nanosheets were initially obtained via a two‐step thermal polymerization approach, consistent with established literature methods [42, 43, 44]. Bulk g‐C_3_N_4_ was first synthesized via thermal polymerization of dicyandiamide at 600 °C for 2 h under an inert atmosphere. The obtained material then underwent a secondary heat treatment at 700 °C for 0.5 h under a slow N_2_ flow, resulting in the formation of exfoliated ultrathin nanosheets. The g‐C_3_N_4_ nanosheets have an approximate thickness of 3 nm, consistent with our previously reported results, indicating that each nanosheet is composed of only a few stacked g‐C_3_N_4_ layers [45]. CsPbI_3_ NCs were prepared through a rapid co‐precipitation process at 190 °C, whereas CsPbI_3_ NWs were produced via a controlled solvothermal method at 170 °C for 10 min. To construct the heterostructures, the as‐prepared CsPbI_3_ NWs were first subjected to a mechano‐chemical mixing step with g‐C_3_N_4_ nanosheets at an optimized composition, enabling preliminary interactions between the surface ligands of the NWs and the functional groups on g‐C_3_N_4_. This was followed by a solvothermal treatment at 150 °C to facilitate interfacial coupling between CsPbI_3_ and g‐C_3_N_4_. During this process, surface ligands on the NWs were effectively removed, promoting their direct and intimate attachment onto the nanosheets. The CsPbI_3_ NCs incorporated composites were fabricated under similar conditions, with detailed procedures described in Supporting Information.

**FIGURE 1:**
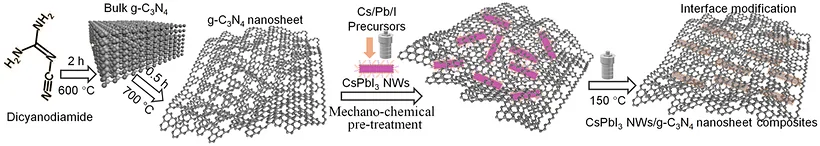
Schematic representation of CsPbI_3_ nanowires assembled with g‐C_3_N_4_ nanosheets to construct the heterostructured system.

Figure 2 displays transmission electron microscopy (TEM) images, revealing the distribution of CsPbI_3_ NCs anchored onto g‐C_3_N_4_ nanosheets. The CsPbI_3_ NCs were closely anchored onto the g‐C_3_N_4_ surface, exhibiting uniform dispersion without noticeable agglomeration (Figure 1a–c). The NCs had an average size of approximately 18 nm and were homogeneously distributed across the nanosheets. This uniform distribution was attributed to the mechano‐chemical pre‐treatment, which promoted an initial interfacial reaction between CsPbI_3_ and g‐C_3_N_4_. Such interaction, mediated by hydrogen bonding between the surface ligands of the NCs and the functional groups of g‐C_3_N_4_, facilitated the even decoration of NCs on the nanosheets surface. As shown in Figure 1d to 1f, most CsPbI_3_ NCs exhibited a well‐defined cubic morphology, with clear lattice fringes and regular geometric features, indicative of high crystallinity and minimal structural defects. High‐resolution TEM images revealed clear lattice fringes with an interplanar spacing of 0.61 nm, which can be assigned to the (100) plane of cubic CsPbI_3_ [21, 22]. To highlight the morphological differences between CsPbI_3_ NCs and NWs before and after integration with g‐C_3_N_4_, their TEM images are provided in Figure S1 (see Supporting Information). After incorporation, the CsPbI_3_ NWs become embedded within the g‐C_3_N_4_ nanosheets, making the original nanowire framework less distinguishable.

**FIGURE 2:**
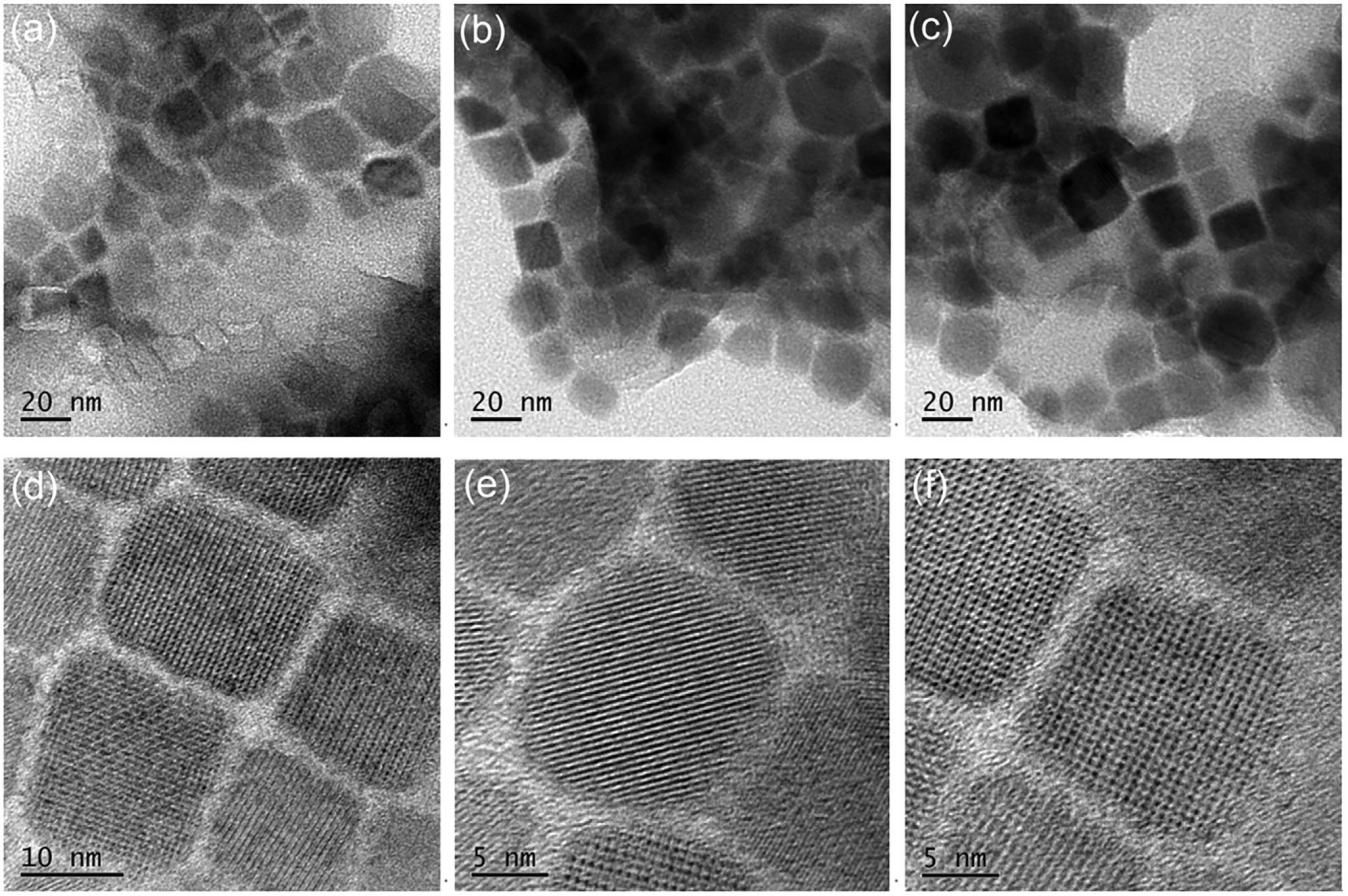
(a to c) Low‐magnification TEM characterization of g‐C_3_N_4_/CsPbI_3_ NCs composites. (d to f) High‐magnification TEM images revealing detailed structural features.

Figure 3 shows the TEM images of the g‐C_3_N_4_/CsPbI_3_ NWs composites synthesized under optimized conditions. High‐aspect‐ratio CsPbI_3_ NWs were homogeneously distributed across the surfaces of g‐C_3_N_4_ nanosheets, forming a continuous and well‐organized NW layer (Figure 3a–c). As shown in Figure 3c, the CsPbI_3_NWswere uniformly and firmly immobilized on the g‐C_3_N_4_ surface in an ordered array‐like arrangement, suggesting that an interfacial pre‐reaction occurred between the nanowires and nanosheets during the mechano‐chemical process. Figures 3d,e further demonstrated that the ultrathin NWs were embedded within the g‐C_3_N_4_ nanosheets rather than being loosely attached, indicating the formation of intimate face‐to‐face interfacial contact. Such an intimate interfacial configuration was conducive to efficient separation and migration of photogenerated charge carriers, thereby enhancing the overall photocatalytic activity. The NWs possess an average diameter of approximately 3.8 nm. High‐resolution TEM (Figure 3f) revealed well‐resolved lattice fringes with an interplanar spacing of 0.31 nm, which could be assigned to the (200) plane of CsPbI_3_, further confirming the preferential growth direction along the 1D nanowire structure. In comparison with the NCs presented in Figure 2, the NWs exhibited relatively reduced crystallinity, which was associated with the controlled slow‐growth conditions, their smaller diameter, and the lower synthesis temperature. To evaluate the elemental distribution within the g‐C_3_N_4_/CsPbI_3_ NWs composites, the corresponding elemental mapping images in Figure 3g–i showed clear signals for Cs, Pb, and I, confirming the successful loading of CsPbI_3_ NWs.

**FIGURE 3:**
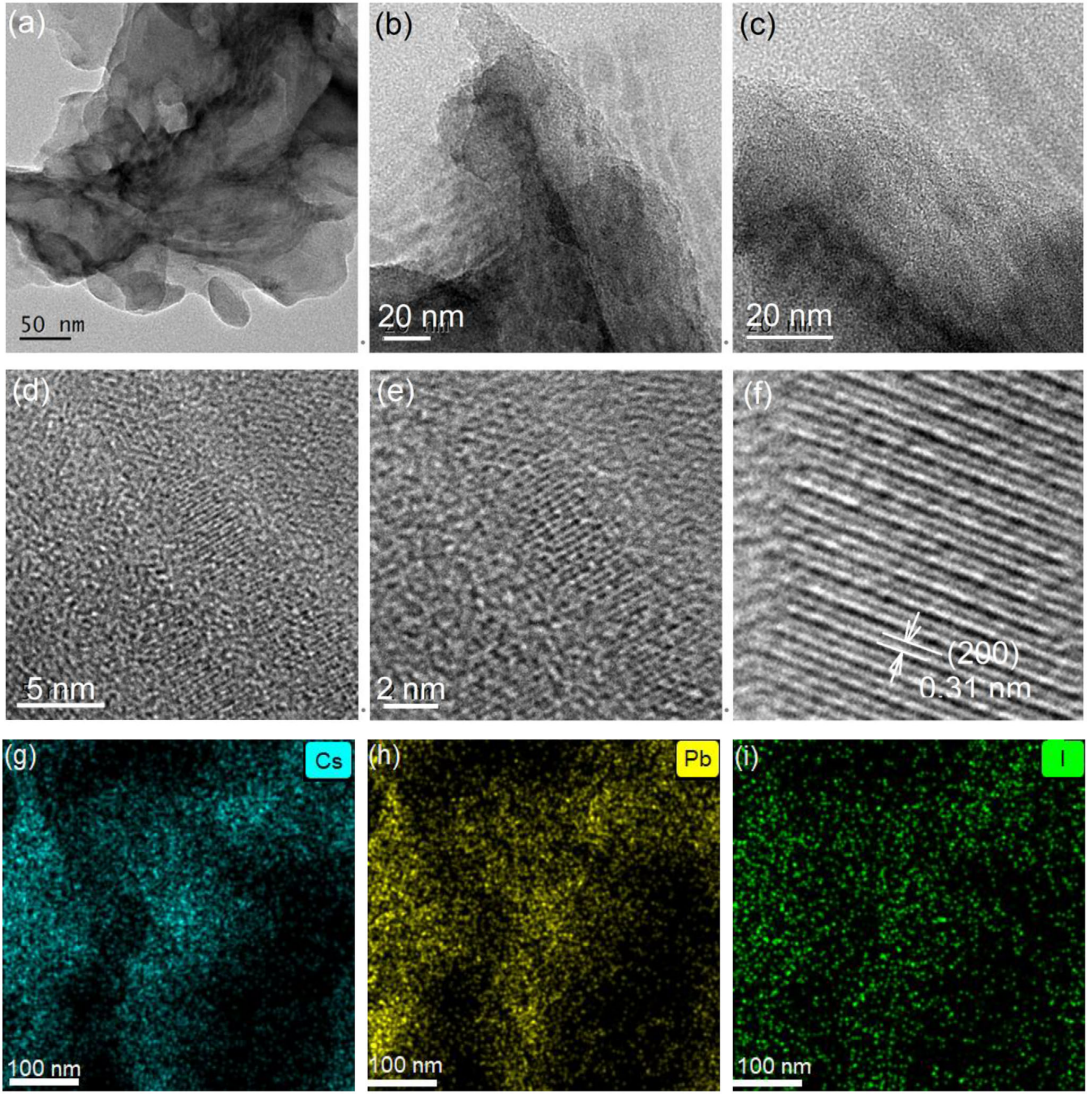
(a to c) Low‐magnification TEM images of g‐C_3_N_4_/CsPbI_3_ NWs composites. (d to f) High‐magnification TEM images of the g‐C_3_N_4_/CsPbI_3_ NWs composites. (g to i) Cs, Pb, and I elemental mapping of sample g‐C_3_N_4_/CsPbI_3_ NWs.

The X‐ray diffraction (XRD) patterns shown in Figure 4a provide crucial evidence for identifying the phase composition of the samples. Distinct diffraction peaks corresponding to both g‐C_3_N_4_ and CsPbI_3_ were observed in the g‐C_3_N_4_/CsPbI_3_ NCs and g‐C_3_N_4_/CsPbI_3_ NWs composite samples. The diffraction peaks located at 14.3°, 20.3°, 28.9°, 32.4°, 35.6°, and 41.3° were indexed to the (100), (110), (200), (211), and (220) planes of cubic CsPbI_3_ (PDF#97‐018‐1288), respectively. The peaks marked with an asterisk in Figure 4a were attributed to g‐C_3_N_4_, with the prominent reflection at 27.5° corresponded to the (002) plane. In comparison to the g‐C_3_N_4_/CsPbI_3_ NCs composites, the g‐C_3_N_4_/CsPbI_3_ NWs sample demonstrated a noticeably diminished intensity of the (110) diffraction peak at 20.3°. This attenuation was associated with the 1D nanowire structure, which altered the preferred crystallographic orientation and consequently affected the diffraction characteristics [46, 47].

**FIGURE 4:**
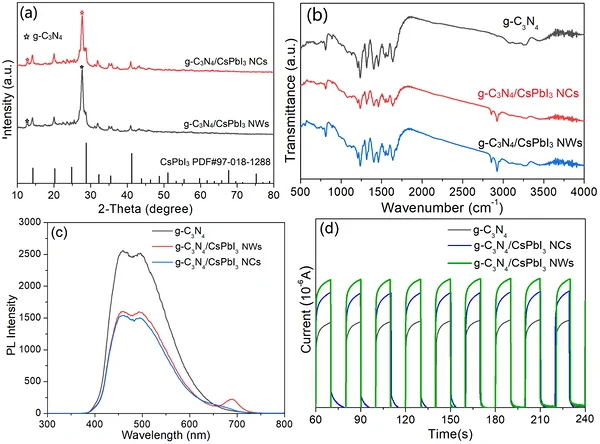
(a) XRD patterns of the CsPbI_3_ NWs and NCs incorporated composite samples. (b) FTIR spectra of the prepared materials. (c)PL spectra of the powder samples. (d) Transient photocurrent response curves collected for the samples.

The Fourier transform infrared (FTIR) spectra shown in Figure 4b highlights the differences between the pristine g‐C_3_N_4_ nanosheets and the composite samples. A characteristic absorption band near 800 cm^−1^ was assigned to the out‐of‐plane bending vibration of the triazine units, while a series of fingerprint bands appear within the 1200–1650 cm^−1^ region. The peaks observed at 1237, 1316, 1406, 1460, 1572, and 1636 cm^−1^ were attributed to the stretching modes of C‐N bonds in the heterocyclic framework [4, 5]. Following incorporation with CsPbI_3_, the characteristic absorption bands within the 1200–1650 cm^−1^ region showed a clear decrease in intensity, suggesting the formation of interfacial interactions between g‐C_3_N_4_ and CsPbI_3_. In addition, new bands appeared at 2851 and 2925 cm^−1^ in the composite systems, which were attributed to the C‐H stretching vibrations of methylene groups derived from oleic acid (OA) and oleylamine (OAm) ligands attached to the surfaces of CsPbI_3_ NCs and NWs. This observation indicated that a small fraction of surface‐bound organic ligands remained after the synthesis process.

Figure 4c shows the photoluminescence (PL) spectra of the fabricated samples. After integrating CsPbI_3_, the defect‐related emission of g‐C_3_N_4_ was significantly attenuated, suggesting more efficient separation of photogenerated charge carriers and suppressed radiative recombination. At the same time, the intrinsic PL signals of CsPbI_3_ NCs and NWs were significantly quenched in the composite systems, although a faint residual emission from the NWs could still be detected. To compare the photophysical behavior before and after integration, the fluorescence decay profiles of the samples are presented in Figure S2 (see Supporting Information). Based on their microstructures and decay characteristics, a two‐exponential fitting model was applied to g‐C_3_N_4_ and CsPbI_3_, whereas a three‐exponential model was used for the g‐C_3_N_4_/CsPbI_3_ NCs (Equation S1 and S2 in Supporting Information). The average lifetimes of CsPbI_3_ NCs, g‐C_3_N_4_, and g‐C_3_N_4_/CsPbI_3_ NCs are 15.41, 16.34, and 26.68 ns, respectively, indicating a clear increase after incorporation. Under photo‐irradiation, the formation of an interfacial built‐in electric field enhanced charge separation, which prolonged the long‐lived decay component associated with surface related processes, while the short‐lived components were suppressed, confirming the successful formation of the heterostructure. Figure 4d displays the transient photocurrent responses of the samples evaluated under light irradiation to further assess charge carrier generation and separation behavior. The introduction of CsPbI_3_ resulted in a pronounced increase in the photocurrent response of g‐C_3_N_4_, indicating enhanced light absorption and more efficient generation of charge carriers. Among all the fabricated samples, the g‐C_3_N_4_/CsPbI_3_ NWs composites exhibited the highest photocurrent density, reflecting outstanding charge separation efficiency and reduced electron‐hole recombination. This improved performance could be attributed to the 1D nanowire architecture, which facilitated directional charge transport and promoted effective charge carrier separation.

X‐ray photoelectron spectroscopy (XPS) was employed to further elucidate the interfacial interaction between CsPbI_3_ and g‐C_3_N_4_, with the g‐C_3_N_4_/CsPbI_3_ NWs composites selected as a representative system. As shown in Figures 5a,b, the N 1s and C 1s spectra of g‐C_3_N_4_ nanosheets were compared before and after integration with CsPbI_3_ NWs. For pristine g‐C_3_N_4_, the N 1s spectrum could be resolved into three main components located at 399.2 eV (pyridinic N, C‐N = C), 400.9 eV (pyrrolic N, N‐(C)_3_), and 401.7 eV (graphitic N). Upon incorporation of CsPbI_3_ NWs, these N 1s peaks shifted slightly toward higher binding energies, whereas the C 1s signals exhibited a corresponding shift to lower binding energies, indicating electronic interaction at the interface between the two components. These changes suggested that the CsPbI_3_ NWs were electronically coupled with g‐C_3_N_4_ through N‐containing functional groups. Figures 5c to 5e present a comparative analysis of the XPS spectra for pristine CsPbI_3_ NWs and the g‐C_3_N_4_/CsPbI_3_ NWs composites. In Figure 5c, the I 3d_5/2_ and I 3d_3/2_ peaks shifted slightly toward higher binding energies, appearing at 618.6 and 630.1 eV, respectively. A similar trend was observed for Cs, where the Cs 3d_5/2_ and Cs 3d_3/2_ peaks moved from 723.7 and 738.0 eV to 724.1 and 738.4 eV, corresponding to an increase of approximately 0.4 eV (Figure 5d). Furthermore, the Pb 4f_7/2_ and Pb 4f_5/2_ signals shifted from 137.8 and 142.9 eV to 138.3 and 143.2 eV, respectively (Figure 5e). These consistent shifts in binding energy provided strong evidence for robust interfacial interactions between g‐C_3_N_4_ and CsPbI_3_. Such behavior was attributed to the interfacial electron redistribution and the establishment of an internal electric field, which was expected to facilitate charge separation and promote charge transfer across the heterojunctions.

**FIGURE 5:**
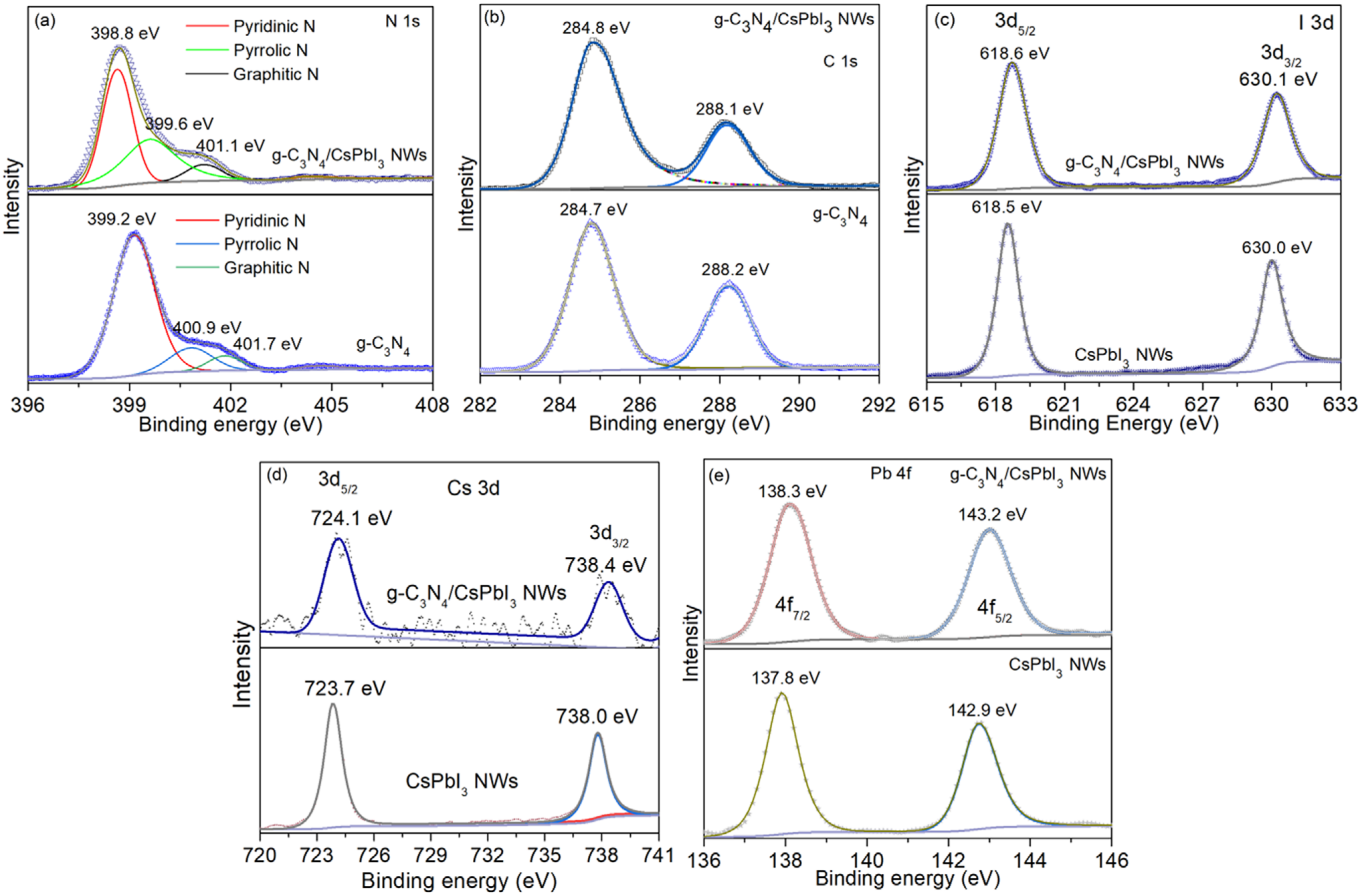
XPS characterization of the samples:(a) N 1s spectra; (b) C 1s spectra; (c) I 3d spectra; (d) Cs 3d spectra; and (e) Pd 4f spectra.

To further elucidate the enhanced charge generation and separation behavior, the absorption and PL spectra of CsPbI_3_ NWs and NCs are presented in Figures 6a,b. Both morphologies exhibited pronounced PL emissions, with maxima centered at 625 nm for the NCs and 687 nm for the NWs. Notably, the NCs displayed a broader emission profile than the NWs, reflecting a wider size distribution, in agreement with the TEM analysis. The observed shift in emission wavelength was attributed to the quantum confinement effects arising from their distinct dimensional characteristics. Furthermore, the different absorption edges observed for the NCs and NWs indicated the differences in their band gap energies. In particular, the red‐shifted absorption edge of the NWs suggested an enhanced ability to harvest visible light compared to the NCs. This shift was accompanied by a reduction in photon energy, reflecting the impact of quantum confinement on their electronic structures. Consequently, these variations in optical and electronic properties resulted in the different photocatalytic performances observed when CsPbI_3_ NCs and NWs were coupled with g‐C_3_N_4_ in composite systems.

**FIGURE 6:**
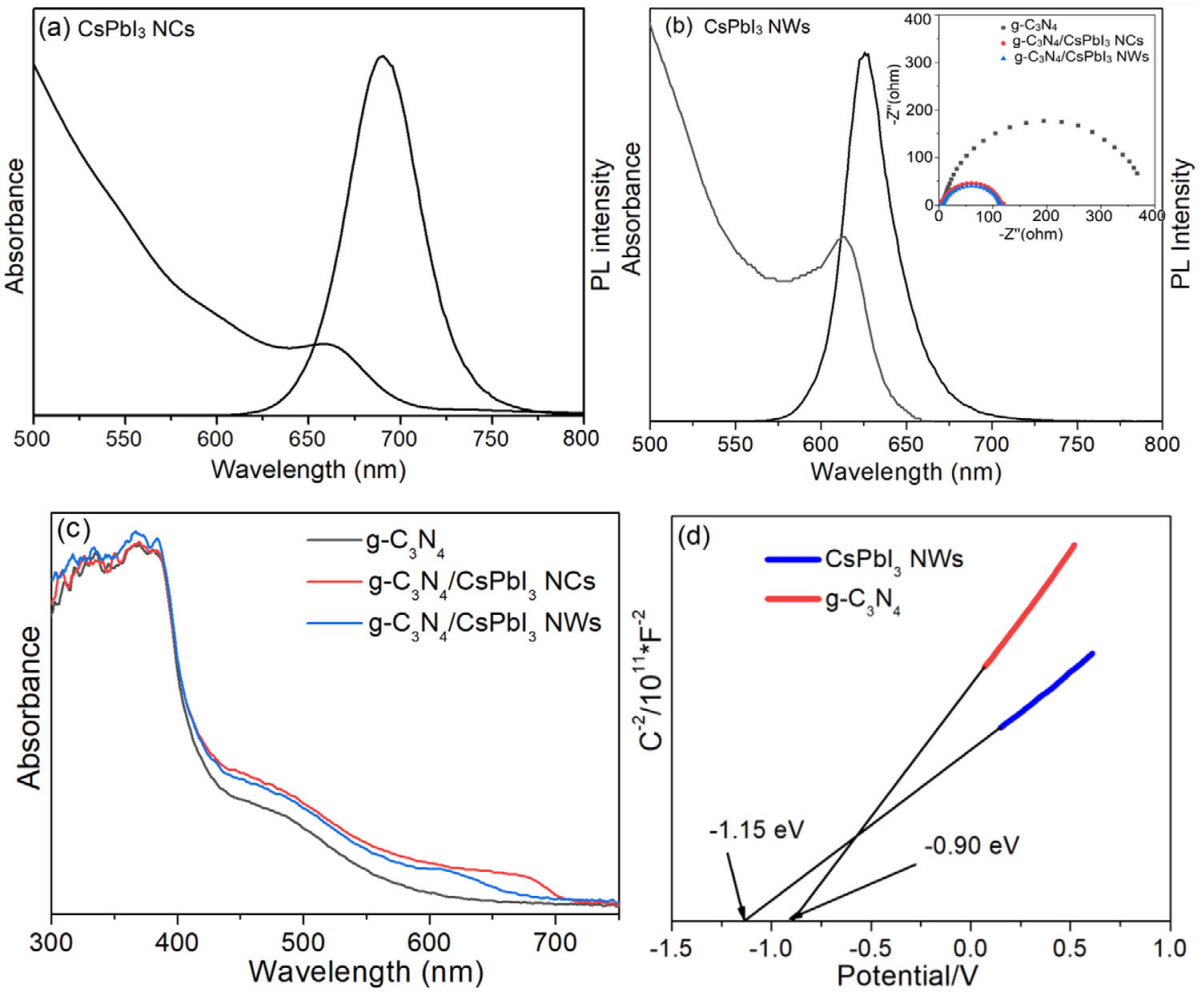
(a) Absorption and PL spectra of CsPbI_3_ NCs measured in solution. (b) Absorption and PL spectra of CsPbI_3_ NWs in solution. (c) UV‐Vis diffuse reflectance spectra of the powder samples. (d) Mott‐Schottky plots of g‐C_3_N_4_ and CsPbI_3_ NWs. The inset in (b) displays the EIS profile of the samples.

Figure 6c displays the UV‐Vis diffuse reflectance spectra of the fabricated powder samples. Upon incorporation of the CsPbI_3_ components, the absorption range was significantly extended into both the visible and red regions. Notably, the g‐C_3_N_4_/CsPbI_3_ NCs composites showed a wider absorption profile than their NWs‐incorporated counterparts. Overall, the composites exhibited significantly improved visible light harvesting capability, as demonstrated by the pronounced red shift in the absorption edge. The optical band gaps (E_g_) were further determined using Tauc plots derived from the diffuse reflectance data in Figure 6c, based on the Kubelka‐Munk function (Equation (1)) [29, 30].

(1) $$A{\left(h\nu -Eg\right)}^{n/2}=\alpha h\nu$$

Here, α, hν, and A denote the absorption coefficient, photon energy, and a proportionality constant, respectively. From the Tauc plot analysis, the band gap energies of g‐C_3_N_4_ and CsPbI_3_ NWs were estimated to be 2.78 and 1.95 eV, respectively. The Mott‐Schottky measurements shown in Figure 6d indicated that CsPbI_3_ NWs possessed a more negative flat‐band potential (‐1.15 V) than g‐C_3_N_4_ nanosheets (‐0.90 V). Based on these values and referenced to the normal hydrogen electrode (NHE), the corresponding conduction band (CB) edge positions were subsequently determined using Equation (2) [10, 11].

(2) $$E_{\mathrm{NHE}\;}=E_{\mathrm{Ag}/\mathrm{AgCl}}\;+0.059*\mathrm{pH}+0.197$$

The valence band (VB) positions were subsequently determined based on the calculated CB potentials. For n‐type semiconductors, the flat‐band potential is commonly considered to approximate the Fermi level [31, 33]. When the two semiconductor components came into contact, electrons were spontaneously transferred from CsPbI_3_ NWs, which possessed a higher Fermi level, to g‐C_3_N_4_ with a lower Fermi level, until thermodynamic equilibrium was achieved. This redistribution of charge at the interfaces led to the formation of a depletion region, wherein the CsPbI_3_ NWs side became electron‐deficient and positively charged, while the g‐C_3_N_4_ side accumulated electrons and acquired a negative charge. As a result, an internal electric field was established across the heterojunctions, which played a pivotal role in promoting efficient charge separation. Moreover, the electrochemical impedance spectroscopy (EIS) measurements (inset of Figure 6b) revealed that both g‐C_3_N_4_/CsPbI_3_ NWs and NCs composites exhibited smaller semicircle radii compared to their individual components, indicating lower charge transfer resistance. This enhanced charge transport behavior could be attributed to the formation of the well‐defined heterostructures with intimate interfacial contact, which facilitated rapid carrier migration and improved overall charge separation efficiency.

g‐C_3_N_4_‐derived heterostructures have attracted substantial attention as platforms for CO_2_ photoreduction and broader solar energy conversion processes. In effective systems, the CB edge of the photocatalyst must be positioned at a more negative potential than the redox levels associated with desired CO_2_ reduction products. For example, typical transformation pathways include the conversion of CO_2_ into CO and CH_4_, as outlined in Equation (3) and (4), which represent key reaction routes commonly discussed in both photocatalytic and electrocatalytic CO_2_ conversion studies.

(3) $$CO_{2}+8H^{+}+8e^{-}\to CH_{4}+2H_{2}OE^{0}=0.17\;Vvs\mathrm{NHE}$$

(4) $$CO_{2}+2H^{+}+2e^{-}\to \mathrm{CO}+H_{2}OE^{0}=-0.11\;Vvs\mathrm{NHE}$$

The reduction of CO_2_ to CO proceeds via a two‐electron transfer pathway, rendering CO generation both thermodynamically and kinetically more favorable. Consequently, product selectivity serves as a crucial criterion for evaluating photocatalytic performance. Among the samples examined, the g‐C_3_N_4_/CsPbI_3_ NWs composites delivered the highest CO production rate (4.6 µmol g^−1^ h^−1^), exceeding that of the NCs‐incorporated counterpart (3.6 µmol g^−1^ h^−1^), while pristine g‐C_3_N_4_ nanosheets exhibited comparatively poor activity. The enhanced performance of the nanowire system suggested that the 1D architecture effectively facilitated charge separation and directional carrier transport. Although the CO evolution rates of the composite samples were lower than those reported for some benchmark systems, their performance remained significant. Importantly, CO was the only detectable reduction product, corresponding to a selectivity of 100%. This exceptional selectivity arose from the well‐matched redox potentials of the photogenerated charge carriers within the g‐C_3_N_4_/CsPbI_3_ heterostructures. Such precise product control is highly advantageous, as separating mixed gaseous products typically requires additional and often complex downstream processes. Consequently, the results hold strong value for both mechanistic studies and future practical implementation. These findings also highlighted the promise of nanowire‐engineered composites as efficient and highly selective platforms for CO_2_ photoreduction.

The cyclic stability of the composites for CO_2_ to CO conversion was evaluated, as shown in Figure 7a. After ten consecutive cycles, the CO production rates of g‐C_3_N_4_/CsPbI_3_ NWs and g‐C_3_N_4_/CsPbI_3_ NCs were 4.2 and 3.4 µmol g^−1^ h^−1^, respectively. These values corresponded to 91% and 94% of their initial activities, demonstrating that both composites exhibited quite high stability, with the NCs‐based sample showing slightly better retention. The XRD pattern of the g‐C_3_N_4_/CsPbI_3_ NWs sample (Figure S3, Supporting Information) remained unchanged after cycling, further confirming the excellent structural stability of the composites.

**FIGURE 7:**
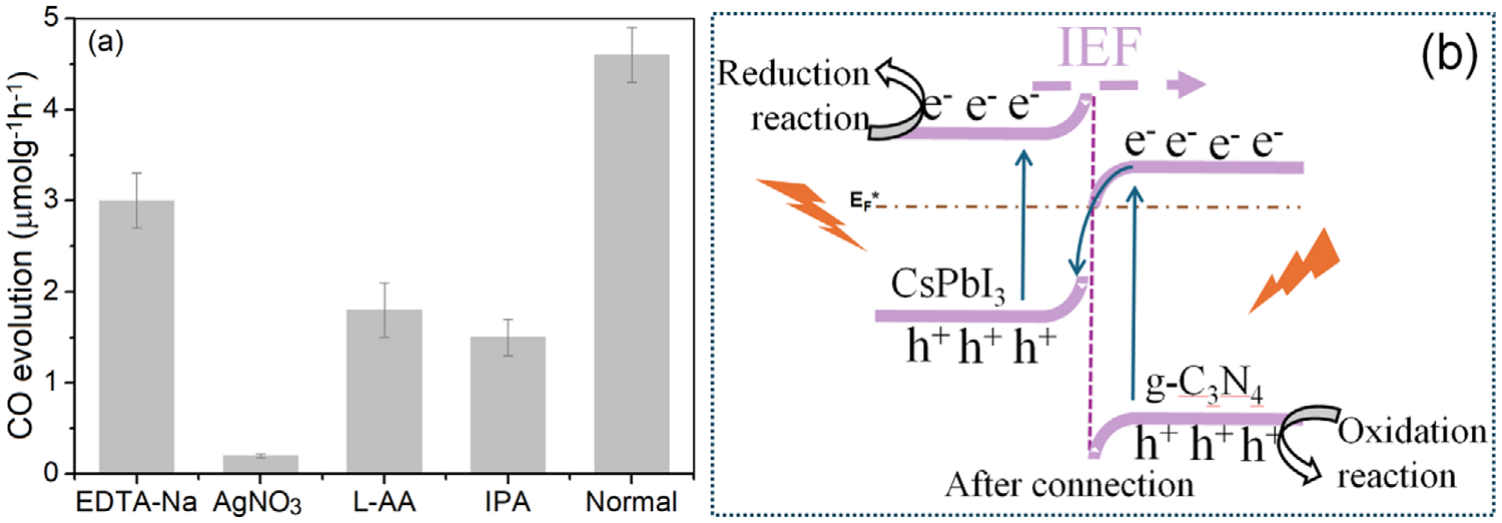
(a) Radical trapping experiments conducted with various scavengers to identify the active species involved in the reaction. (b) Schematic illustration depicting the separation and migration of photogenerated charge carriers following the integration of CsPbI_3_ components with g‐C_3_N_4_ nanosheets.

To evaluate the potential cytotoxicity associated with Pb^2+^ release, the concentration of Pb ions in the solution after the stability test was measured to be 0.31 nM. This extremely low level was attributed to the small amount of CsPbI_3_ incorporated into the composites. In addition, the hydrophobic organic ligands on the CsPbI_3_ surface further suppressed Pb^2+^ leaching and contributed to the overall stability of the catalysts. To clarify the underlying photocatalytic pathway, a series of radical scavenging experiments were performed using ethylenediaminetetraacetic acid disodium salt (EDTA‐2Na), AgNO_3_, L‐ascorbic acid (L‐AA), and isopropanol (IPA) to selectively capture holes (h^+^), electrons (e^−^), superoxide radicals (^•^O_2_^−^), and hydroxyl radicals (^•^OH), respectively. As presented in Figure 7a, the CO generation rate was significantly suppressed in the presence of L‐AA and IPA, indicating that electron‐related species, particularly ^•^O_2_^−^ radicals, played a decisive role in the CO_2_ reduction process. This suggested that photogenerated electrons were actively involved in activating dissolved O_2_ to form reactive superoxide intermediates. Based on these observations, an S‐scheme charge transport mechanism was proposed, as illustrated in Figure 7b. Owing to the distinct electronic structures of g‐C_3_N_4_ and CsPbI_3_, interfacial charge redistribution occurred upon contact, resulting in Fermi level realignment and the establishment of an internal electric field across the heterojunctions. This process induced opposite band bending in the two components (i.e., g‐C_3_N_4_ and CsPbI_3_). Under light irradiation, electrons excited in the CB of g‐C_3_N_4_ migrated toward the VB of CsPbI_3_, where they recombined with the photogenerated holes. Therefore, electrons with strong reducing power were preserved in the CB of CsPbI_3_, while highly oxidative holes accumulated in the VB of g‐C_3_N_4_. The spatial separation of these charge carriers effectively maintained their redox potential, thereby facilitating efficient photocatalytic reactions. In this system, reduction reactions were primarily driven by the electrons in CsPbI_3_, whereas oxidation processes were governed by the holes residing in g‐C_3_N_4_. EPR spectroscopy was further used to identify the reactive radical species, as shown in Figure S4 (see Supporting Information). The characteristic multi‐peak pattern of DMPO‐^•^OH confirmed the formation of hydroxyl radicals (^•^OH), while the distinct multi‐peak signal of DMPO‐^•^O_2_^−^ indicated the presence of superoxide radicals (^•^O_2_^−^). These results demonstrated that both ^•^OH and ^•^O_2_^−^ participate as active species during the photocatalytic processes.

The fabricated layered face‐to‐face heterostructures exhibited outstanding performance in photocatalytic NO oxidation, primarily owing to their enlarged specific surface area and the high density of exposed active sites. Within this system, the oxidative strength of the photogenerated charge carriers was a decisive factor governing activity and reaction pathways. In contrast to CO_2_ reduction, which was predominantly photoinduced electron driven, NO abatement was largely mediated by reactive oxygen species, particularly ^•^O_2_^−^ and ^•^OH radicals. The formation of ^•^O_2_^−^ originated from the reduction of adsorbed O_2_ by photogenerated electrons (Equation (5)). These superoxide species subsequently initiated the oxidation of NO, producing intermediate compounds such as NO_2_ and N_2_O_5_ through stepwise reactions (Equation (8) and (9)). In parallel, ^•^OH radicals were generated via consecutive transformations involving ^•^O_2_^−^ and H_2_O_2_ (Equation (6) and (7)). These highly oxidative ^•^OH species further reacted with both NO and NO_2_, driving their conversion into more stable end products, including HNO_3_ and HNO_2_ (Equation (10) and (11)).

(5) $$e^{-}+O_{2}\to^{•}{O_{2}}^{-}$$

(6)

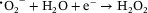



(7)

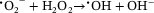

(8) $$\mathrm{NO}{+}^{•}{O_{2}}^{-}\to NO_{2}$$

(9) $$NO_{2}{+}^{•}{O_{2}}^{-}\to N{O_{3}}^{-}$$

(10) $$\mathrm{NO}{+}^{•}\mathrm{OH}\to \mathrm{HN}O_{2}$$

(11) $$NO_{2}{+}^{•}\mathrm{OH}\to \mathrm{HN}O_{3}$$

The photogenerated electrons in the CB of CsPbI_3_ transferred to dissolved O_2_, generating ^•^O_2_^−^ radicals that initiate the oxidation of NO to NO_2_. Concurrently, the surfaces of g‐C_3_N_4_ effectively adsorbed H_2_O molecules, which subsequently participated in secondary reactions with both photogenerated electrons and ^•^O_2_^−^ to form H_2_O_2_. This intermediate further decomposed into highly oxidative ^•^OH radicals. These ^•^OH species acted as the dominant reactive intermediates, driving the sequential oxidation of NO and NO_2_ into stable end products such as HNO_2_ and HNO_3_. In parallel, the presence of abundant surface functionalities (e.g., ‐NH_2_ and ‐OH groups) on g‐C_3_N_4_ promoted strong adsorption of NO molecules, facilitating interfacial reaction kinetics and enhancing overall photocatalytic efficiency. Considering the high toxicity of NO_2_, it is essential to achieve efficient NO conversion while suppressing NO_2_ accumulation. As presented in Figure 8a, the NO removal efficiencies of pristine g‐C_3_N_4_, g‐C_3_N_4_/CsPbI_3_ NWs, and g‐C_3_N_4_/CsPbI_3_ NCs were 23.9%, 59.2%, and 69.5%, respectively, demonstrating the pronounced enhancement induced by CsPbI_3_ incorporation, particularly in the NCs incorporated composites. Stability assessments (Figure 8b) further revealed that the g‐C_3_N_4_/CsPbI_3_ NCs catalyst retained its initial activity over five successive reaction cycles, demonstrating robust structural and functional durability. Moreover, NO_2_ selectivity (Figure 8c) was significantly reduced from 14.2% for g‐C_3_N_4_ to 5.1% and 5.0% for the NWs and NCs composites, respectively. In this case, NO reacted with ^•^O_2_^−^ to form NO_2_. When ^•^O_2_^−^ was generated in excess, the produced NO_2_ could undergo further oxidation by ^•^O_2_^−^ to yield NO_3_^−^, reducing the amount of NO_2_. In the g‐C_3_N_4_/CsPbI_3_ heterostructure, the S‐scheme charge transfer pathway promoted abundant ^•^O_2_^−^ formation, which in turn led to the observed low NO_2_ selectivity. Notably, the g‐C_3_N_4_/CsPbI_3_ NCs system simultaneously delivered the highest NO removal efficiency and the lowest NO_2_ selectivity, underscoring its superior capability for efficient and selective photocatalytic NO oxidation.

**FIGURE 8:**
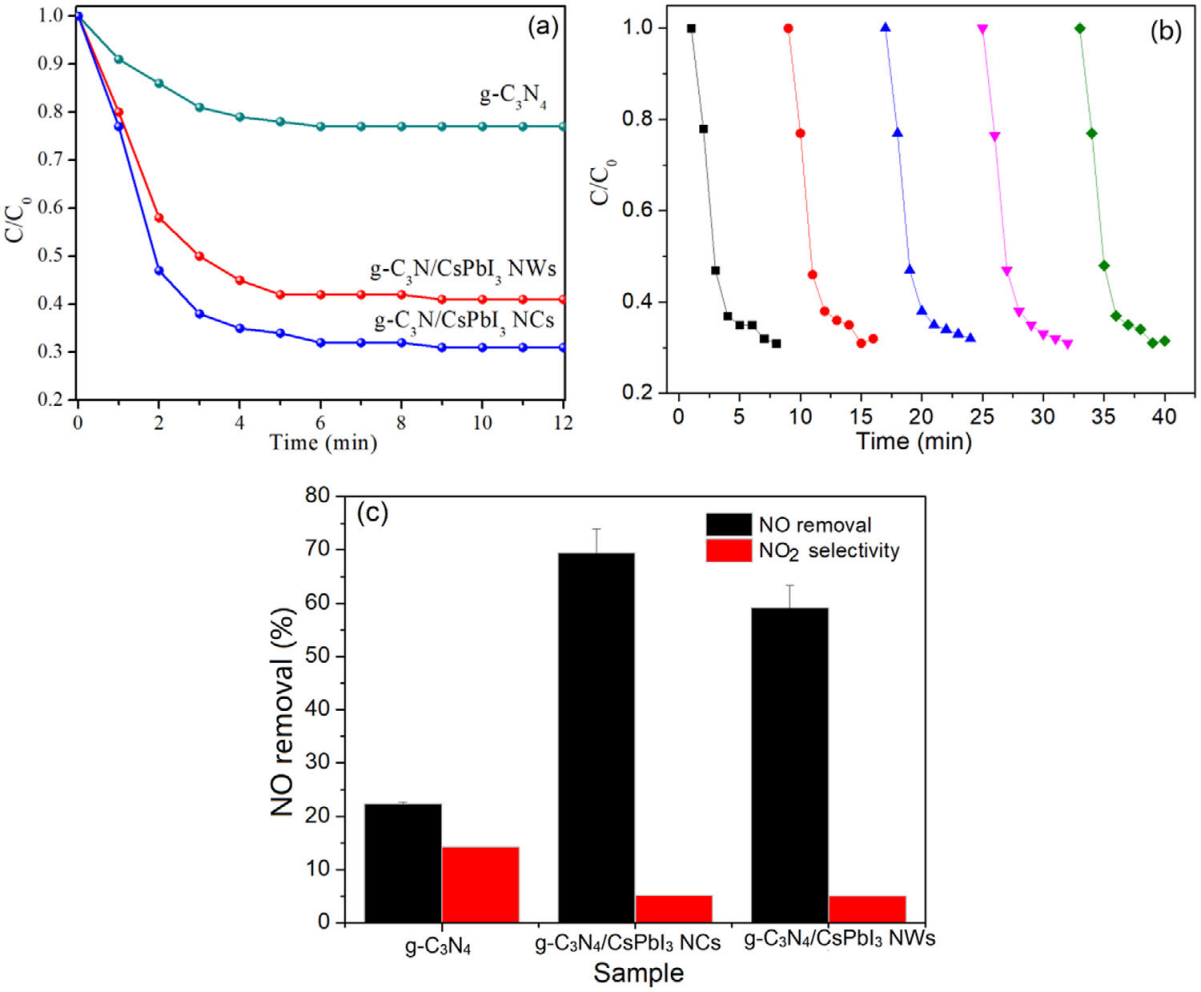
(a) Photocatalytic NO removal efficiency profiles of the prepared samples. (b) Cyclic durability assessment of NO removal performance over g‐C_3_N_4_/CsPbI_3_ NCs. (c) Comparative analysis of NO conversion rates and NO_2_ selectivity among different samples.

As illustrated in Figure 1, hydrophobic CsPbI_3_ nanostructures capped with organic ligands were first combined with g‐C_3_N_4_ nanosheets through a mechano‐chemical process. The mechanical treatment promoted partial stripping of surface ligands from CsPbI_3_, while simultaneously enabling strong interfacial contact with g‐C_3_N_4_ via hydrogen bonding interactions. Subsequent solvothermal processing further strengthened the interfacial coupling, leading to the formation of a robust and well‐integrated heterostructure. Within this system, an S‐scheme charge transport mechanism governed the carrier dynamics: photo‐excited electrons in the CB of g‐C_3_N_4_ recombined with holes in the VB of CsPbI_3_, thereby retaining highly reducing electrons in the CB of CsPbI_3_ and strongly oxidizing holes in the VB of g‐C_3_N_4_. The accumulated electrons on CsPbI_3_ effectively activated molecular oxygen to generate ^•^O_2_^−^, which played a dominant role in driving NO oxidation. Consequently, the synergistic effect of intimate interfacial contact, favorable band alignment, directional charge migration, and suppressed electron‐hole recombination significantly enhanced the photocatalytic redox capability, resulting in outstanding NO removal performance coupled with minimized NO_2_ formation.

Perovskite nanostructures exhibit fundamentally different physicochemical behavior compared with their bulk analogues, largely governed by pronounced quantum confinement effects. As the dimensional scale decreases, the exciton binding energy increases and can surpass thermal energy at ambient conditions, thereby reinforcing the redox strength of photoexcited charge carriers. Consequently, key optoelectronic parameters (including band structure, light absorption characteristics, and oxidation capability) can be systematically modulated through precise control over particle size and morphology. In the case of CsPbI_3_ NCs, an increase in particle size led to a red shift in PL emission and a gradual narrowing of the bandgap. For instance, previously reported values indicated a reduction in bandgap from 1.85 eV (6.6 nm) to 1.73 eV (12.6 nm), highlighting the influence of quantum confinement [21]. In the present study, CsPbI_3_ NCs possess an average size of approximately 18 nm, whereas the NWs exhibit a much smaller diameter of ∼3.8 nm. Correspondingly, the estimated bandgaps for the NCs and NWs are ∼1.68 eV and ∼1.95 eV, respectively. The narrower bandgap of the NCs enables more effective absorption in the red‐light region, thereby enhancing solar energy utilization. In addition, nanoscale dimensions strongly influenced phase stability. Smaller CsPbI_3_ quantum dots (e.g., 6.6‐8.4 nm) can stabilize the cubic phase through increased surface energy contributions [21]. However, the larger NCs synthesized here retained a well‐defined cubic morphology characterized by clear lattice fringes and highly ordered crystal facets (Figure 2), indicative of high crystallinity and relatively low defect density compared with the ultrathin NWs (Figure 3). In general, increasing particle size reduced the density of surface and structural defects, which in turn improved PL efficiency and facilitated more efficient charge transport. According to our previously published work, CsPbI_3_ NCs possess well‐defined facets, whereas CsPbI_3_ NWs contain a significantly higher density of surface defects such as vacancies, dangling bonds, and adsorbed ions [15, 16, 27]. These defect sites can trap photogenerated charge carriers, reducing their separation efficiency and ultimately lowering the NO removal performance. In contrast, the g‐C_3_N_4_/CsPbI_3_ NCs composites exhibited outstanding photocatalytic NO removal performance. This enhancement could be attributed to a combination of stronger oxidative capability, improved charge transport dynamics, reduced recombination losses, and broadened light absorption.

## 3 Conclusion

Face‐to‐face g‐C_3_N_4_/CsPbI_3_ heterostructures were constructed through a two‐step strategy involving an initial mechano‐chemical pre‐treatment followed by solvothermal processing. The mechanical pre‐reaction promoted partial detachment of surface ligands from CsPbI_3_, enabled direct and uniform anchoring of the perovskite components onto g‐C_3_N_4_ nanosheets. Subsequent solvothermal treatment strengthened interfacial coupling, resulting in a robust heterostructure with an S‐scheme charge transfer configuration. This rational interfacial engineering, together with broadened light absorption, significantly enhanced the photocatalytic redox capacity of the composites. Importantly, photocatalytic behavior could be tuned by controlling the morphology of the incorporated CsPbI_3_ components. The NWs incorporated heterostructures exhibited outstanding CO_2_ reduction activity, delivering a CO production rate of 4.6 µmol g^−1^ h^−1^ with 100% selectivity. This superior reduction performance arose from the 1D architecture of the NWs, which promoted directional charge transport and effectively suppressed electron‐hole recombination. In contrast, the g‐C_3_N_4_/CsPbI_3_ NCs system demonstrated excellent photocatalytic oxidation capability, achieving 69.5% NO removal at an initial concentration of 600 ppb, accompanied by a low NO_2_ selectivity of 5.0%. This behavior arose from the CsPbI_3_ NCs, which generate high‐energy photogenerated electron‐hole pairs that provided strong driving forces for surface redox reactions. This effect was further amplified by the S‐scheme heterostructure, which preserved highly oxidative holes and sustained efficient oxidation processes. Collectively, this work established a versatile strategy for designing g‐C_3_N_4_‐based CsPbI_3_ modified heterostructures with tunable architectures and optimized interfacial properties, offering significant promise for both solar driven fuel production and environmental purification applications.

## Author Contributions

**Xiao Zhang**: conceptualization, methodology, data curation, investigation, formal analysis, project administration, funding acquisition, writing – original draft, writing – review and editing. **San Ping Jiang**: conceptualization, methodology, data curation, writing – review and editing, investigation.

## Conflicts of Interest

The authors declare no conflicts of interest.

## Supporting information




**Supporting File**: advs78119‐sup‐0001‐SuppMat.docx.

## Data Availability

The data that support the findings of this study are available from the corresponding author upon reasonable request.

## References

[1] X. Zhang and S. P. Jiang , “Recent Advances in G‐C_3_N_4_Nanoarchitectonics for Efficient Photocatalytic H_2_O_2_ Evolution,” Coordination Chemistry Reviews 560 (2026): 217889, 10.1016/j.ccr.2026.217889.

[2] H. Lü , D. Wang , P. Yang , and X. Zhang , “WO_3_ Implanted Pt‐g‐C_3_N_4_ Nanosheets towards Boosted Photocatalytic Tetracycline Hydrochloride Removal and Water Splitting,” Journal of Environmental Chemical Engineering 13, no. 5 (2025): 118685, 10.1016/j.jece.2025.118685.

[3] J. G. Cuadra , N. P. F. Gonçalves , K. B. Tayeb , et al., “Surface‐Functionalized G‐C_3_N_4_/3D‐Printed Alkali‐Activated Materials for the Removal of Emerging Water Pollutants,” Chemical Engineering Journal 529 (2026): 172457, 10.1016/j.cej.2025.172457.

[4] X. Zhang and S. P. Jiang , “Cu/Au Nanoparticles Embedded in G‐C_3_N_4_ Nanosheets Toward Efficient Photocatalytic CO_2_/NO Conversion and H_2_ Generation,” Journal of Environmental Chemical Engineering 14, no. 2 (2026): 121853, 10.1016/j.jece.2026.121853.

[5] H. Lü , W. Shi , P. Yang , and X. Zhang , “MoO_x_ Embedded S‐g‐C_3_N_4_ Frameworks With Enhanced Photocatalytic H_2_ Generation and Phenol Removal,” Environmental Research 287 (2025): 123123, 10.1016/j.envres.2025.123123. 41093001

[6] X. Zhang , X. Ji , and P. Yang , “MoSe_2_ Nanosheets Horizontally Implanted on Co‐N‐Doped Carbon Nanotubes via Mo‐N Bonding for Overall Water Splitting,” Applied Surface Science 723 (2026): 165666, 10.1016/j.apsusc.2025.165666.

[7] X. Zhang , “Transition Metal Dichalcogenide Catalysts Incorporating Hollow Carbon Spheres Toward Water Splitting and Supercapacitors,” Chemical Communications 62, no. 18 (2026): 5141–5157, 10.1039/d5cc07364b. 41664901

[8] X. Zhang , B. Xu , and P. Yang , “CuSe Nanoplates Horizontally Implanted on C‐Doped G‐C_3_N_4_ Nanosheets for H_2_O Splitting and Highly Selective CH_4_ Conversion,” Journal of Alloys and Compounds 1057 (2026): 186771, 10.1016/j.jallcom.2026.186771.

[9] X. Zhang and P. Yang , “Enhanced Charge Carrier Separation and Transfer in G‐C_3_N_4_and Tungsten Oxide/Sulfide Composite Photocatalysts,” Physical Chemistry Chemical Physics 27, no. 47 (2025): 25254–25265, 10.1039/d5cp03846d. 41250957

[10] X. Zhang , C. Jia , Y. Xue , and P. Yang , “Fabrication of rGO/g‐C_3_N_4_ Composites via Electrostatic Assembly Towards Charge Separation Control,” RSC Advances 7, no. 69 (2017): 43888–43893, 10.1039/C7RA07706H.

[11] D. Wang , X. Zhang , H. Zhang , P. Song , and P. Yang , “Effect of Precursors on Cu Particle Distribution in G‐C_3_N_4_ Nanosheets towards Efficient Photocatalytic Degradation and H_2_ Generation,” International Journal of Hydrogen Energy 68 (2024): 463–471, 10.1016/j.ijhydene.2024.04.198.

[12] C. Xie , X. Zhang , H. S. Chen , and P. Yang , “Highly Bright and Stable CsPbX_3_@Cs_4_PbX_6_ Hexagonal Nanoarchitectonics Created by Controlling Dissolution‐Recrystallization of CsPbX_3_ Nanomaterials,” Small 20, no. 43 (2024): 2403648, 10.1002/smll.202403648. 38881372

[13] W. Shi , X. Zhang , C. Xie , H. S. Chen , and P. Yang , “Growth of CsPbX_3_ Nanocrystals Using sol–gel SiO_2_ Solid Powders as Reactors Without Capping Agents Towards Anomalous Stable Emission Membranes,” Chemical Engineering Journal 493 (2024): 152638, 10.1016/j.cej.2024.152638.

[14] C. Xie , X. Zhang , W. Shi , and P. Yang , “Highly Luminescent CsPbX_3_@MIL‐53(Al) Nanoarchitectonics With Anomalous Stability towards Flexible Emitting Films,” Journal of Alloys and Compounds 986 (2024): 174132, 10.1016/j.jallcom.2024.174132.

[15] W. Shi , X. Zhang , C. Xie , H. S. Chen , and P. Yang , “Blue Emitting CsPbBr_3_: High Quantum Confinement Effect and Well‐Adjusted Shapes (Nanorods, Nanoplates, and Cubes) Toward White Light Emitting Diodes,” Advanced Optical Materials 12, no. 7 (2024): 2302129, 10.1002/adom.202302129.

[16] W. Shi , X. Zhang , H. S. Chen , S. P. Jiang , and P. Yang , “Bright Self‐Trapped Exciton Emission of CsPbBr_3_@CsPb_2_Br_5_ Nanostructures Created by Dissolution and Recrystallization of Hydrophilic Cu:CsPbBr_3_ ,” Small 22, no. 39 (2026): 73956, 10.1002/smll.73956. PMC13360731 42186950

[17] W. Shi , P. Yang , and X. Zhang , “Sr Derived Synthesis of CsPbX_3_ Nanocrystals for Extraordinary Stable Luminescence,” Applied Surface Science 730 (2026): 166325, 10.1016/j.apsusc.2026.166325.

[18] D. Wang , X. Zhang , W. Shi , and P. Yang , “Glass‐Encapsulated CsPbBr_3_ Nanocrystals Incorporated With Hollow Bowl TiO_2_ Nanoarrays towards Efficient NO Removal,” Journal of Environmental Chemical Engineering 13, no. 1 (2025): 115146, 10.1016/j.jece.2024.115146.

[19] W. Shi , P. Yang , and X. Zhang , “Enhanced Stability and Brightness for Small CsPbX_3_ Nanocrystals via Solvent and Ligand Controlling,” Materials Today Physics 58 (2025): 101877, 10.1016/j.mtphys.2025.101877.

[20] W. Shi , X. Zhang , and P. Yang , “In Situ Heteroepitaxial Growth of CsPbX_3_@CsPb_2_X_5_ Core–Shell Nanocrystals in Zeolite Framework Toward Extraordinary Brightness and Stability,” Advanced Optical Materials 13, no. 26 (2025): 01395, 10.1002/adom.202501395.

[21] H. Ebe , R. Suzuki , S. Sumikoshi , et al., “Guanidium Iodide Treatment of Size‐Controlled CsPbI_3_ Quantum Dots for Stable Crystal Phase and Highly Efficient Red LEDs,” Chemical Engineering Journal 471 (2023): 144578, 10.1016/j.cej.2023.144578.

[22] W. Shi , Y. Tian , P. Yang , and X. Zhang , “CsPbI_3_/Pt‐g‐C_3_N_4_ Heterostructures Toward Efficient Photocatalytic Dichlorophenol Removal,” Surfaces and Interfaces 92 (2026): 109359, 10.1016/j.surfin.2026.109359.

[23] W. Shi , X. Zhang , Y. Tian , and P. Yang , “Aqueous Glass‐Coated CsPbI_3_ Quantum Dots Implanted in G‐C_3_N_4_ Nanosheets for Efficient Photocatalytic Water Splitting and H_2_O_2_Generation,” Journal of Environmental Chemical Engineering 12, no. 5 (2024): 114018, 10.1016/j.jece.2024.114018.

[24] W. Shi , X. Zhang , and P. Yang , “Ligand Derived Slow Growth and Passivation of Small CsPbX_3_ Nanocrystals With High Brightness and Stable Tunable Emitting,” Materials Today Chemistry 46 (2025): 102752, 10.1016/j.mtchem.2025.102752.

[25] W. Shi , X. Zhang , and P. Yang , “Blue‐Emitting CsPbBr_3_ Nanocrystals: Synthesis Progress and Bright Photoluminescence,” Langmuir 41, no. 9 (2025): 5762–5781, 10.1021/acs.langmuir.4c05108. 40008989

[26] W. Shi , P. Yang , and X. Zhang , “Mercapto Derived CsPbX_3_ Confinement Growth in Metal‐Organic Frameworks Towards Extraordinary Brightness and Stability,” Materials Today Chemistry 43 (2025): 102462, 10.1016/j.mtchem.2024.102462.

[27] C. Xie , X. Zhang , H. S. Chen , and P. Yang , “Synthesis‐Kinetics of Violet‐ and Blue‐Emitting Perovskite Nanocrystals With High Brightness and Superior Stability Toward Flexible Conversion Layer,” Small 20, no. 19 (2024): 2308896, 10.1002/smll.202308896. 38057136

[28] C. Liu , P. Yang , and X. Zhang , “MoS_2_ Nanosheets Grown Homogeneously on Hollow Carbon Spheres for Efficient Hydrogen Evolution Reaction and Supercapacitor,” International Journal of Hydrogen Energy 97 (2025): 1348–1356, 10.1016/j.ijhydene.2024.12.008.

[29] C. Jia , X. Zhang , and P. Yang , “Construction of Anatase/Rutile TiO_2_ Hollow Boxes for Highly Efficient Photocatalytic Performance,” Applied Surface Science 430 (2018): 457–465, 10.1016/j.apsusc.2017.06.163.

[30] C. Li , Y. Tian , S. Wang , P. Yang , and X. Zhang , “Ag_2_Se Nanoparticles Anchored on S‐g‐C_3_N_4_ Nanosheets towards Efficient Photocatalytic Tetracycline Hydrochloride Removal and H_2_ Generation,” Journal of Environmental Chemical Engineering 12, no. 5 (2024): 114170, 10.1016/j.jece.2024.114170.

[31] T. Song , X. Zhang , Q. Che , and P. Yang , “Heterojunction Nanoarchitectonics With SnS_2_/G‐C_3_N_4_ S‐Scheme Toward Enhanced Photooxidation and Photoreduction,” Journal of Industrial and Engineering Chemistry 113 (2022): 389–400, 10.1016/j.jiec.2022.06.014.

[32] Z. Yang , X. Xu , Z. Li , et al., “Leveraging Polaron Effect for Solar‐Driven Efficient Peroxymonosulfate Activation in Water Purification,” Angewandte Chemie International Edition 64, no. 22 (2025): 202503901, 10.1002/anie.202503901. 40139965

[33] J. Teng , W. Li , Z. Wei , et al., “Coupling Photocatalytic Hydrogen Production With Key Oxidation Reactions,” Angewandte Chemie International Edition 63, no. 50 (2024): 202416039, 10.1002/anie.202416039. 39301679

[34] M. Li , X. Zhang , S. Lu , and P. Yang , “Phase Transformation, Morphology Control, and Luminescence Evolution of Cesium Lead Halide Nanocrystals in the Anion Exchange Process,” RSC Advances 6, no. 105 (2016): 103382–103389, 10.1039/C6RA22070C.

[35] F. Xu , K. Meng , B. Cheng , S. Wang , J. Xu , and J. Yu , “Unique S‐Scheme Heterojunctions in Self‐Assembled TiO_2_/CsPbBr_3_ Hybrids for CO_2_ Photoreduction,” Nature Communications 11, no. 1 (2020): 4613, 10.1038/s41467-020-18350-7. PMC7490390 32929077

[36] H. Liao , J. Zheng , Y. Huang , et al., “Dual Active Sites Based CsPbBr_3_/Porous Carbon Nitrides S‐Scheme Heterojunction for Synergetic Vanillin and Hydrogen Peroxide Production,” Chemical Engineering Journal 524 (2025): 169068, 10.1016/j.cej.2025.169068.

[37] M. Ou , W. Tu , S. Yin , et al., “Amino‐Assisted Anchoring of CsPbBr_3_ Perovskite Quantum Dots on Porous g‐C_3_N_4_ for Enhanced Photocatalytic CO_2_ Reduction,” Angewandte Chemie International Edition 57, no. 41 (2018): 13570–13574, 10.1002/anie.201808930. 30155982

[38] J. Cai , J. Huang , S. Wang , et al., “Crafting Mussel‐Inspired Metal Nanoparticle‐Decorated Ultrathin Graphitic Carbon Nitride for the Degradation of Chemical Pollutants and Production of Chemical Resources,” Advanced Materials 31, no. 15 (2019): 1806314, 10.1002/adma.201806314. 30697837

[39] J. Zhang , C. D. Sewell , H. Huang , and Z. Lin , “Closing the Anthropogenic Chemical Carbon Cycle Toward a Sustainable Future via CO_2_ Valorization,” Advanced Energy Materials 11, no. 47 (2021): 2102767, 10.1002/aenm.202170186.

[40] S. He , Z. Hu , C. He , et al., “Nanoconfinement‐Enabled Resistance to Water Matrix Components for Selective Degradation of Micropollutants,” Angewandte Chemie International Edition 64, no. 14 (2025): 202424827, 10.1002/anie.202424827. 39824787

[41] S. He , Y. Chen , J. Fang , Y. Liu , and Z. Lin , “Optimizing Photocatalysis via Electron Spin Control,” Chemical Society Reviews 54, no. 5 (2025): 2154–2187, 10.1039/d4cs00317a. 39838850

[42] C. Wang , X. Zhang , Y. Zhu , K. Zhu , X. Luan , and P. Yang , “Growth in Situ of NiSe_2_/Ni Hybrids in N‐Doped Carbon Nanotubes for Enhanced Overall Water Splitting,” Electrochimica Acta 454 (2023): 142386, 10.1016/j.electacta.2023.142386.

[43] X. Zhang and P. Yang , “Tungsten Oxide/G‐C_3_N_4_ Heterostructures: Composition, Structure, and Photocatalytic Applications,” Langmuir 41, no. 11 (2025): 7191–7211, 10.1021/acs.langmuir.4c05287. 40079178

[44] X. Zhang , P. Wang , P. Yang , and S. P. Jiang , “Photo‐Chemical Property Evolution of Superior Thin G‐C_3_N_4_ Nanosheets With Their Crystallinity and Pt Deposition,” International Journal of Hydrogen Energy 45, no. 41 (2020): 21523–21531, 10.1016/j.ijhydene.2020.06.031.

[45] T. Song , X. Zhang , and P. Yang , “Interface Engineering of W_2_C/W_2_N co‐Catalyst on G‐C_3_N_4_ Nanosheets for Boosted H_2_ Evolution and 4‐Nitrophenol Removal,” Environmental Science: Nano 9, no. 5 (2022): 1888–1899, 10.1039/d2en00104g.

[46] Z. Wen , W. Zhai , C. Liu , et al., “Controllable Synthesis of CsPbI_3_ Nanorods With Tunable Photoluminescence Emission,” RSC Advances 9, no. 43 (2019): 24928–24934, 10.1039/C9RA04600C. PMC9069944 35528692

[47] W. Shi , X. Zhang , H. S. Chen , and P. Yang , “Zn‐Derived Ligand Engineering Towards Stable and Bright CsPbI_3_ Nanocrystals for White Emitting,” Journal of Materials Chemistry C 10, no. 36 (2022): 13117–13123, 10.1039/D2TC02995B.

