# Supplementary material for: Perovskite CsPbI3 Nanocrystal and Nanowire Incorporated G‐C3N4 Nanosheets for CO2 Reduction and NO Removal
Source: Adv Sci (Weinh). 2026 Sep 30:e78119. Online ahead of print. doi: 10.1002/advs.78119 (PMC13627998; doi:10.1002/advs.78119)
Supplement: Supplementary file 1 [file ADVS-9999-e78119-s001.docx]

Supporting Information

Perovskite CsPbI_3_ nanocrystal and nanowire incorporated g-C_3_N_4_ nanosheets for CO_2_ reduction and NO removal

Xiao Zhang^a^*, San Ping Jiang^b^

Prof. X. Zhang

Advanced Institute for Materials Research (WPI-AIMR), Tohoku University, Sendai 980-8577, Japan.

E-mail: zhang.xiao.d8@tohoku.ac.jp

Prof. S. P. Jiang

WA School of Mines: Minerals, Energy and Chemical Engineering, Curtin University, Perth WA 6845, Australia.

Experimental Section

**1. Chemicals**

Lead(II) iodide (PbI_2_, 99.9%) and p‑benzoquinone (p‑BQ, 99%) were procured from Aladdin Biochemical Technology Co., Ltd. Cesium carbonate (Cs_2_CO_3_, 99.9%), oleic acid (OA, 90%), 1‑octadecene (ODE, 90%), and oleylamine (OAm, 90%) were supplied by Sigma Aldrich. n‑Hexane (C_6_H_14_, 99%) and dimethyl sulfoxide (DMSO, 99%) were obtained from Tianjin Fuyu Fine Chemical Co., Ltd. Chloroplatinic acid hexahydrate (H_2_PtCl_6_·6H_2_O, 99%), dicyandiamide, L‑histidine (L‑His, 99%), isopropanol (IPA, 99%), and potassium persulfate (K_2_S_2_O_8_, 97%) were purchased from Sinopharm Chemical Reagent Co., Ltd. Ethylenediaminetetraacetic acid disodium salt dihydrate (EDTA‑2Na, 98%), tetracycline hydrochloride (99%), and 2,4‑dichlorophenol (98%) were provided by Shanghai Macklin Biochemical Co., Ltd.

**2. Synthesis of the samples**

Ultrathin g-C_3_N_4_ nanosheets were prepared through a sequential two-step thermal polymerization process. First, bulk g-C_3_N_4_ was synthesized by heating dicyandiamide at 600 ℃ for 2 h under a nitrogen atmosphere. The obtained yellow product was then subjected to a secondary thermal treatment at 700 ℃ for 30 min under the same inert conditions to induce exfoliation into thinner layers. After naturally cooling to ambient temperature, the material was finely ground and extensively washed with deionized water and ethanol to remove residual impurities and any unreacted precursors.

CsPbI_3_ NWs and NCs were synthesized using a modified solvothermal method. For the preparation of NWs, cesium and lead precursors were first generated separately. The cesium oleate (Cs-OA) precursor was formed by reacting Cs_2_CO_3_ with OA in ODE at 150 ℃ under a nitrogen atmosphere until complete dissolution. Meanwhile, the lead precursor solution was obtained by dissolving PbI_2_ in ODE with the addition of OA and OAm under inert conditions. Following precursor preparation, the Cs-OA solution was swiftly introduced into the Pb-containing solution, and the resulting mixture was transferred into a sealed autoclave. The solvothermal reaction was carried out at 170 ℃ for 10 min to induce the formation of CsPbI_3_ NWs. After completion, the product was purified via a two-step centrifugation procedure. The reaction mixture was first centrifuged at 10,000 rpm to isolate the solid product, which was then re-dispersed in n‑hexane and subjected to a second centrifugation at 5,000 rpm. This process effectively removed residual impurities and yielded a stable dispersion of purified CsPbI_3_ NWs.

For the synthesis of CsPbI_3_ NCs, a hot injection method was employed. Initially, 0.5 mmol of Cs_2_CO_3_ was loaded into a three-neck flask, followed by the addition of 2.5 mL OA and 6 mL ODE. The mixture was degassed under a nitrogen atmosphere and heated to 120 ℃ for 1 h to remove residual moisture and oxygen. The temperature was then increased to 150 ℃ and maintained briefly until complete dissolution of the Cs_2_CO_3_, yielding a clear Cs-oleate precursor solution. This solution was subsequently kept at 100 ℃ for further use. In parallel, PbI_2_ was dissolved in 6 mL ODE in a four-neck flask and heated to 120 ℃ under a continuous N_2_ flow for 1 h. After complete dissolution, a ligand mixture consisting of OA, OAm, and 1-dodecanethiol was introduced. The reaction temperature was then raised to 190 °C and maintained for 5 min to ensure precursor stabilization. Subsequently, 0.4 mL of the pre-heated Cs-oleate solution was rapidly injected into the Pb precursor mixture. After a brief reaction period of approximately 5 s, the reaction was immediately quenched using an ice water bath to terminate crystal growth. The obtained CsPbI_3_ NCs were collected after cooling to room temperature and further purified to remove residual reactants and excess ligands.

g-C_3_N_4_/CsPbI_3_ NWs and g-C_3_N_4_/CsPbI_3_ NCs heterostructures were prepared through a combined mechano-chemical treatment followed by solvothermal processing. In a typical synthesis, 50 mg of g-C_3_N_4_ nanosheets were used as the substrates. Subsequently, 2 mL of the CsPbI_3_ NWs dispersion was gradually added dropwise onto the g-C_3_N_4_ powder, followed by continuous grinding until complete evaporation of the solvent. This grinding-drying process was further repeated with the addition of 1 mL of ODE to promote uniform mixing and interfacial contact, and the procedure was conducted for a total of five cycles to ensure effective integration of the two components. The resulting homogeneous powder was then dispersed in ethylene glycol and transferred into a sealed autoclave, where it was heated at 150℃ for 2 h to obtain the final heterostructures. The use of ethylene glycol enhanced the hydrophilicity of the composites, improving adsorption toward target gas molecules. For comparison, g-C_3_N_4_/CsPbI_3_ NCs composites were synthesized using an identical procedure, with the CsPbI_3_ NWs dispersion replaced by the corresponding NCs solution. The loading amounts of CsPbI_3_ (NWs or NCs) were systematically optimized, and the best-performing composition is reported in this study. To evaluate the influence of CsPbI_3_ loading, the amounts of CsPbI_3_ NCs and NWs were systematically adjusted and their photocatalytic performance was compared. The optimized composition identified through this screening is presented in the manuscript.

**3. Characterization**

A combination of complementary characterization methods was employed to comprehensively elucidate the structural, compositional, and optical features of the prepared materials. The crystalline phase and structural ordering were analyzed by XRD using a Bruker D8 Advance diffractometer. FTIR spectroscopy was utilized to identify surface functional groups and bonding environments. The elemental composition and surface chemical states were further examined by XPS (Thermo ESCALAB 250Xi). Morphological features and microstructural characteristics were investigated using high-resolution transmission electron microscopy (HR-TEM, JEOL JEM-2100F). EDS mapping of the composite samples was carried out using high‑angle annular dark field scanning TEM (HAADF‑STEM). Optical absorption behavior was measured with a UV-Vis spectrophotometer (Hitachi U-4100), while steady-state PL spectra were collected on a Hitachi F-4600 spectrophotometer under excitation at 365 nm. The concentration of free Pb ions in the solution was quantified using an inductively coupled plasma mass spectrometer (ICP‑MS; Finnigan Element 2, Thermo Fisher Scientific) to assess Pb release after the cyclic stability test. Free radical trapping experiments were conducted using a Bruker A300 electron spin resonance (ESR) spectrometer. Fluorescence decay curves were recorded using a single photon counting spectrofluorometer (JY‑IBH, Horiba) with an excitation wavelength of 370 nm. The average lifetimes of g-C_3_N_4_ and CsPbI_3_ NCs were determined using a two‑exponential fitting model (Eq. S1), whereas those of the g-C_3_N_4_/CsPbI_3_ NCs composite were obtained using a three‑exponential model (Eq. S2). In each case, τ_ave_ represents the average lifetime calculated from the individual decay components τ_i_ and their corresponding amplitudes B_i_.

$$\tau_{ave}=\frac{B_{1}{\tau_{1}}^{2}+B_{2}{\tau_{2}}^{2}}{B_{1}\tau_{1}+B_{2}\tau_{2}} (S1)$$

$$\tau_{ave}=\frac{B_{1}{\tau_{1}}^{2}+B_{2}{\tau_{2}}^{2}+B_{3}{\tau_{3}}^{2}}{B_{1}\tau_{1}+B_{2}\tau_{2}+B_{3}\tau_{3}} (S2)$$

**4. CO_2_ reduction**

For photocatalytic CO_2_ reduction tests, 20 mg of catalyst was dispersed in 15 mL of deionized water to obtain a uniform suspension. Before light irradiation, the reactor was purged by alternating cycles of high-purity CO_2_ injection and vacuum evacuation for 15 min to remove residual air and achieve CO_2_ saturation. The photocatalytic reactions were carried out under visible light illumination using a 300 W xenon lamp (PLS-SXE 300, Perfect Light, China) fitted with a 420 nm cut-off filter. The light intensity within the 420-780 nm range was calibrated to 100 mW cm^-2^ using a radiometer (FZ-A, China). To ensure thermal stability, the system temperature was maintained at 20 ℃ through a circulating water-cooling setup. During the reaction, the suspension was continuously stirred at 200 rpm and exposed to light for 4 h. Gaseous products were collected at regular intervals and quantified using gas chromatography (GC-7920).

**5. NO removal**

Photocatalytic NO oxidation performance was evaluated using a custom-designed reaction system. In a typical experiment, 100 mg of the catalyst was uniformly dispersed onto a culture dish and placed inside a cylindrical reactor sealed with a quartz window. A stream of NO gas was diluted with air to achieve an initial concentration of 600 ppb and then introduced into the reactor at a flow rate of 1200 sccm. The photocatalytic reaction was carried out under irradiation from a 200 W xenon lamp, while a circulating water system was employed to maintain the reaction temperature at ambient conditions. The variation in NO concentration during the reaction was continuously monitored in real time using a nitrogen oxide analyzer (Thermo Fisher Scientific). For safety and environmental protection, the outlet gas was passed through an alkaline NaOH solution to remove residual NO_x_ before being released.

**6. Photoelectrochemical measurements**

Photoelectrochemical measurements were performed using a standard three-electrode system in a 0.1 M Na_2_SO_4_ aqueous electrolyte. The working electrode was fabricated by immobilizing the catalyst onto indium tin oxide (ITO) conductive glass. In a typical procedure, 2.5 mg of catalyst was dispersed in a mixed solution of 500 μL ethanol and 8 μL Nafion, followed by ultrasonication to obtain well-dispersed ink. An aliquot of 120 μL of this suspension was then drop-cast onto the ITO substrate and allowed to dry under ambient conditions, yielding a stable photoelectrode. The photoelectrochemical properties were systematically investigated through transient photocurrent measurements, EIS, and Mott-Schottky analysis to assess charge separation behavior, interfacial charge transfer dynamics, and semiconductor characteristics.

Figure S1. TEM images of the samples: (a) g-C_3_N_4_/CsPbI_3_ NCs. (b) CsPbI_3_ NCs. (c) g-C_3_N_4_/CsPbI_3_ NWs. (b) CsPbI_3_ NWs.

Figure S2. Fluorescence decay plots of the samples.

Figure S3. XRD pattern of g-C_3_N_4_/CsPbI_3_ NWs after cyclic stability test.

Figure S4. EPR analysis of active radicals in sample g-C_3_N_4_/CsPbI_3_ NCs.
